# Supplementary material for: Ultraconserved bacteriophage genome sequence identified in 1300-year-old human palaeofaeces
Source: Nat Commun. 2024 Jan 23;15:495. doi: 10.1038/s41467-023-44370-0 (PMC10805732; doi:10.1038/s41467-023-44370-0)
Supplement: Supplementary file 1 — Supplementary Information [file 41467_2023_44370_MOESM1_ESM.pdf]

# Ultraconserved bacteriophage genome sequence identified in 1300-year-old human palaeofaeces

Rozwalak P., Barylski J., Wijesekara Y., Dutilh B.E., Zielezinski A.

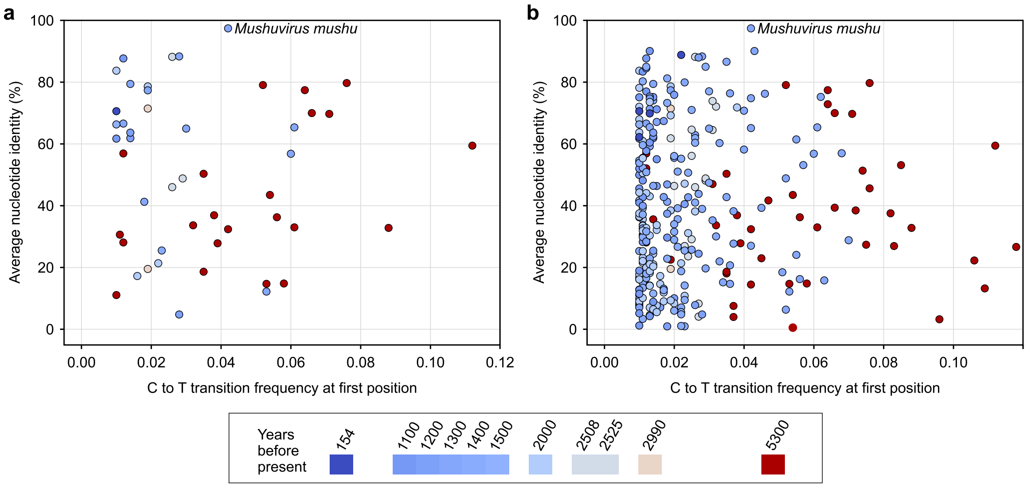


**Supplementary Figure 1. Relation between average nucleotide identity and damage patterns of ancient bacteriophage genomes. a** 49 complete genomes **b** all 298 ancient genomes. Point colors represent the age of samples. Data underlying the scatterplots are available in Supplementary Data 3, 6, and 7. Source data are provided as a Source Data file.

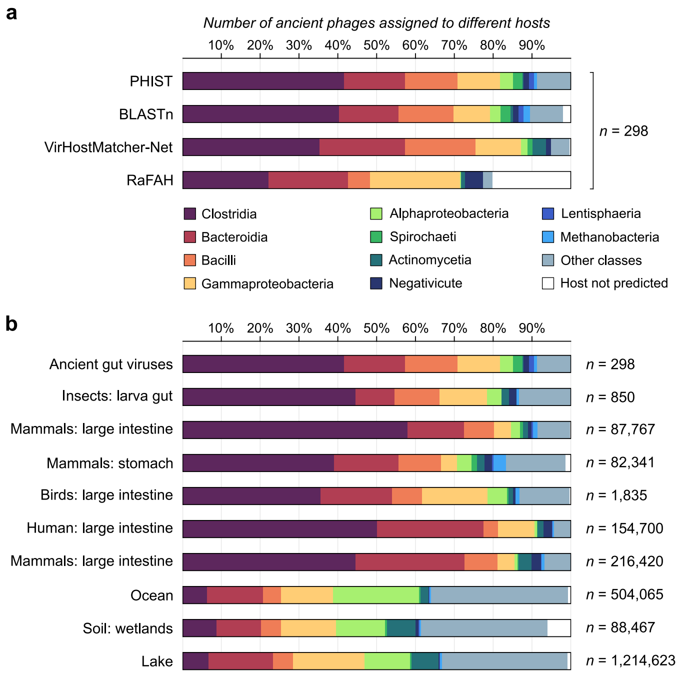


**Supplementary Figure 2. Bacteria classes linked to ancient and contemporary viruses from IMG/VR.**

**a** Comparison of host predictions for 298 ancient metagenomic gut viruses (aMGVs) using four tools BLASTn[1], PHIST[2], VirHostMatcher-Net[3], and RaFAH[4]. **b** The distribution of different classes of host bacteria assigned to ancient viruses and hosts of viruses from different ecosystems in IMG/VR[5].

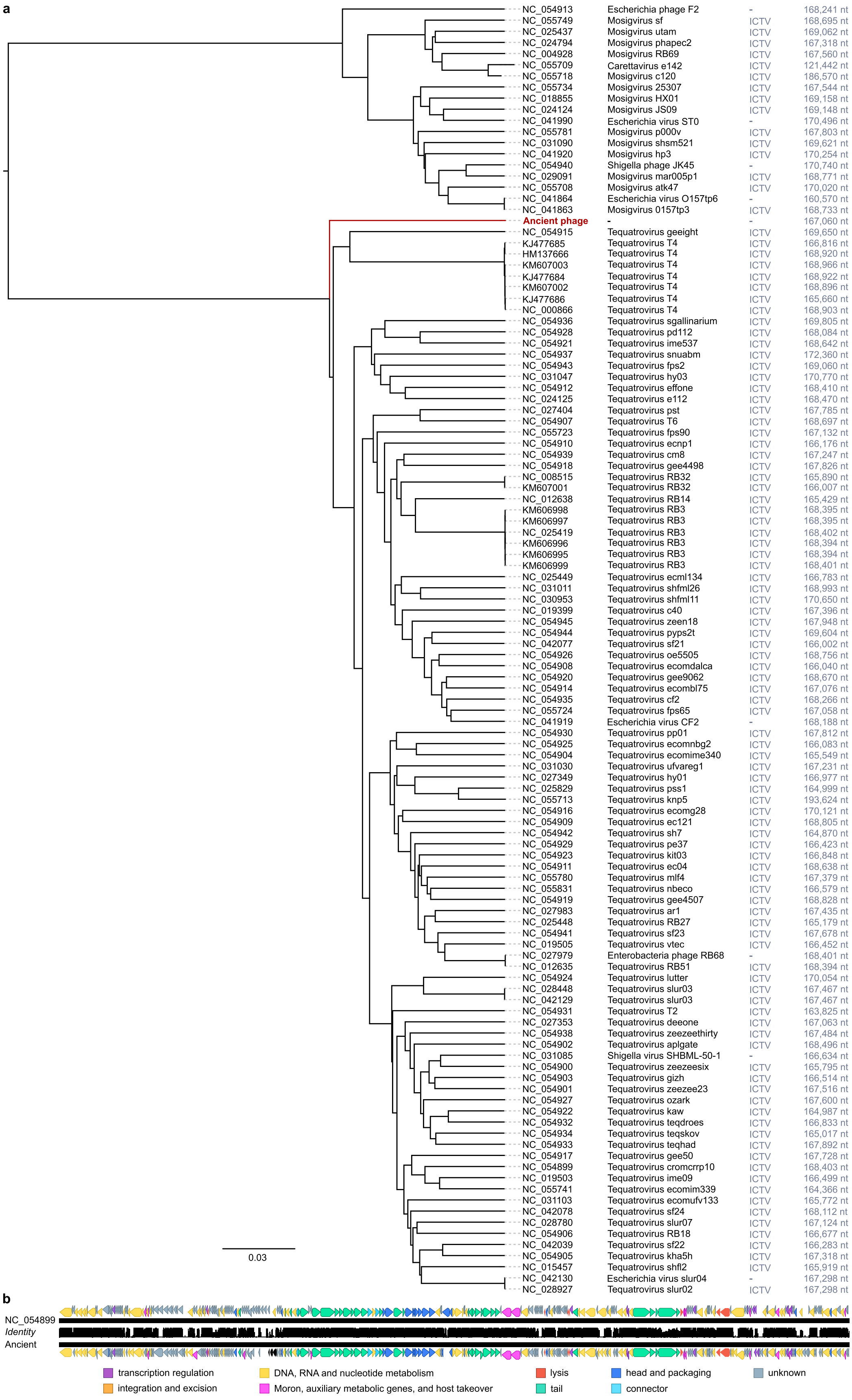


**Supplementary Figure 3. Ancient bacteriophage related to contemporary *Tequatrovirus* species.**

**a** Fragment of the proteomic tree created by the VipTree[6] server for the ancient T4-related phage genome (NODE_1_length_167060_cov_116.590443). **b** Comparison of sequence conservation and gene organization between the ancient T4-related phage genome and modern reference *Tequatrovirus* genome (NC_054899) with the highest average nucleotide identity (88.12%).

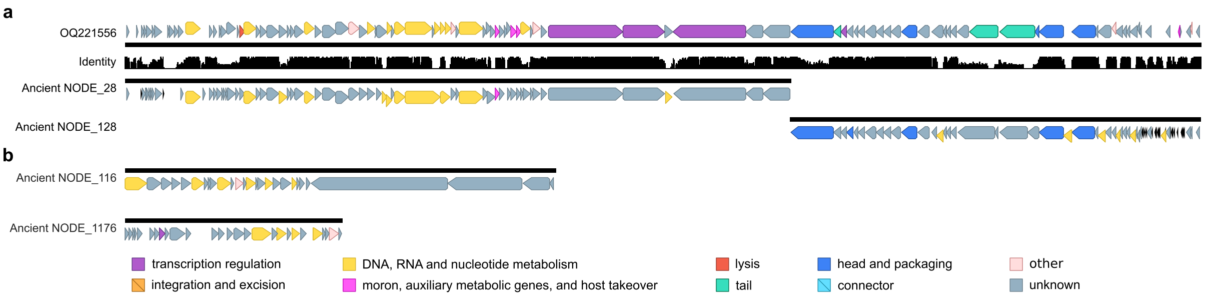


**Supplementary Figure 4. Four ancient bacteriophage contigs related to contemporary *Crassvirales*.**

**a** Comparison of two ancient contigs and *Bacteroides* phage PhiCrAssBcn21 (GenBank accession: OQ221556) – their closest modern relative. Annotation of modern reference was retrieved from the original GenBank record (accession: OQ221556), and ancient contigs were annotated by phage_contig_annotator pipeline with PHROGs v.4 (https://github.com/Yasas1994/phage_contig_annotator). **b** Structure of two genome fragments with no close modern relatives in GenBank and RefSeq.

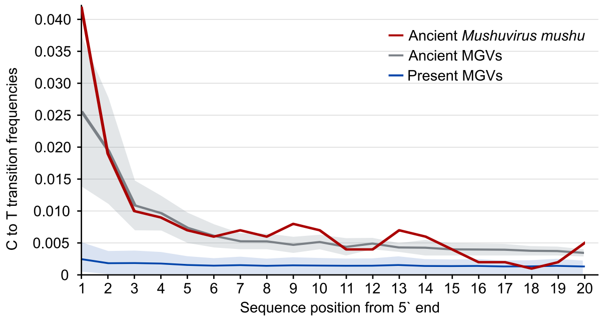


**Supplementary Figure 5. Damage patterns in ancient and contemporary virus genomes.** Comparison of damage patterns between ancient *Mushuvirus mushu*, other ancient metagenomic gut viruses (aMGVs), and modern viral genomes. The solid line shows the mean frequency of C → T substitutions, and the shaded areas indicate the standard deviation. Source data are provided as a Source Data file.

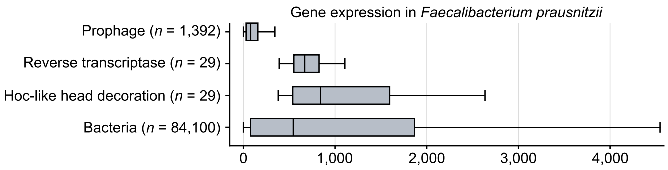


**Supplementary Figure 6. Gene expression in cultured *Faecalibacterium prausnitzi* from 29 RNA-seq samples[7].** Hoc-like head decoration and reverse transcriptase genes are expressed at a comparable level to bacterial genes and more than other phage genes. Source data are provided as a Source Data file.

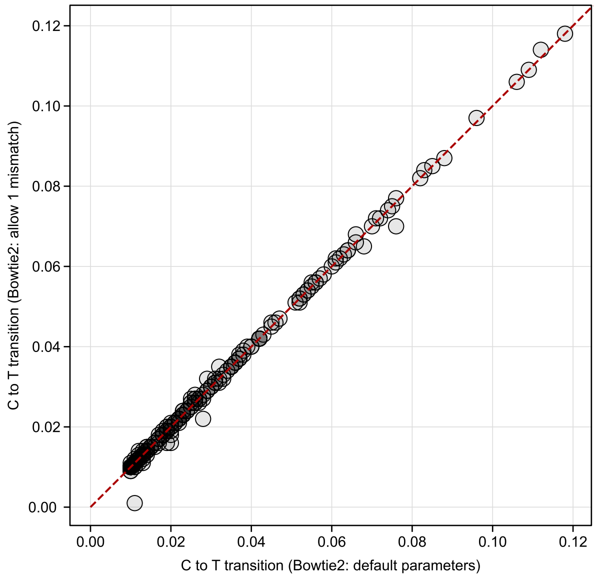


**Supplementary Figure 7. Comparison of damage patterns of 298 ancient phage genomes between two variants of Bowtie2 mapping, default and sensitive.** The damage patterns are shown as C → T transition frequencies at the first position of sequencing reads mapping to ancient phage genomes. Source data are provided as a Source Data file.

## Supplementary References

1. Altschul, S. F., Gish, W., Miller, W., Myers, E. W. & Lipman, D. J. Basic local alignment search tool. *J Mol Biol* **215**, 403–410 (1990).
2. Zielezinski, A., Deorowicz, S. & Gudyś, A. PHIST: fast and accurate prediction of prokaryotic hosts from metagenomic viral sequences. *Bioinformatics* **38**, 1447–1449 (2022).
3. Wang, W. *et al.* A network-based integrated framework for predicting virus–prokaryote interactions. *NAR Genom Bioinform* **2**, 1–19 (2020).
4. Coutinho, F. H. *et al.* RaFAH: Host prediction for viruses of Bacteria and Archaea based on protein content. *Patterns* **2**, 100274 (2021).
5. Camargo, A. P. *et al.* IMG/VR v4: an expanded database of uncultivated virus genomes within a framework of extensive functional, taxonomic, and ecological metadata. *Nucleic Acids Res* **51**, D733–D743 (2023).
6. Nishimura, Y. *et al.* ViPTree: the viral proteomic tree server. *Bioinformatics* **33**, 2379–2380 (2017).
7. Auger, S. *et al.* Gene co-expression network analysis of the human gut commensal bacterium Faecalibacterium prausnitzii in R-Shiny. *PLoS One* **17**, e0271847 (2022).
