# Supplementary material for: Ultraconserved bacteriophage genome sequence identified in 1300-year-old human palaeofaeces
Source: Nat Commun. 2024 Jan 23;15:495. doi: 10.1038/s41467-023-44370-0 (PMC10805732; doi:10.1038/s41467-023-44370-0)
Supplement: Supplementary file 3 — Description of Additional Supplementary Files [file 41467_2023_44370_MOESM3_ESM.pdf]

Description of Additional Supplementary Files

File Name: Supplementary Data 1
Description: Information about samples used in our study of ancient phages (from https://github.com/SPAAM-community/AncientMetagenomeDir).

File Name: Supplementary Data 2
Description: Identification of 298 ancient metagenomic gut viruses (aMGVs) with three tools (Jaeger AA, VirSorter2, and VIBRANT).

File Name: Supplementary Data 3
Description: Information about 298 ancient metagenomic gut viruses (aMGVs) including CheckV, ANI clustering and PyDamage results. *p*-values were obtained from the PyDamage tool. Specifically, *p*-value is calculated from the likelihood-ratio test-statistic using a chi-squared distribution, and q-value defines *p*-value corrected for multiple testing using Benjamini-Hochberg procedure.

File Name: Supplementary Data 4
Description: Classification of 298 ancient metagenomic gut viruses (aMGVs) based on their predicted lifestyles. Lifestyle predictions were made using a combination of BLASTn searches against the Unified Human Gastrointestinal Genome (UHGG) bacterial metagenomes and BACPHLIP predictions. aMGVs were considered temperate if they aligned with a bacterial genome for at least 50% of their length or if BACPHLIP predicted them as temperate. Those not meeting these criteria were classified as virulent.

File Name: Supplementary Data 5
Description: Details about vContact network (Fig. 2) and viral clusters. Viral clusters consist of ancient and modern viruses from IMG/VR v.4 (high-confidence genomes only) and NCBI RefSeq or GenBank using an accession from ICTV Virus Metadata Resources (VMR_20-190822_MSL37.2, created 08/31/2022). *P*-values were obtained from vContact2 output.

File Name: Supplementary Data 6
Description: Comparison of nucleotide and amino acid sequence similarity between 298 ancient phages and contemporary phages from GenBank and RefSeq. Each ancient phage is paired with a contemporary phage showing the highest average nucleotide identity (ANI), calculated using VIRIDIC, and average amino acid identity (AAI), calculated using EzAAI. Host information is provided from NCBI.

File Name: Supplementary Data 7
Description: Comparison of nucleotide and amino acid sequence similarity between 298 ancient phages and contemporary phages from IMG/VR. Each ancient phage is paired with a contemporary phage showing the highest average nucleotide identity (ANI), calculated using VIRIDIC, and average amino acid identity (AAI), calculated using EzAAI. Host and biome information is provided from IMG/VR.

File Name: Supplementary Data 8
Description: Host assignments of 298 ancient metagenomic gut viruses (aMGVs) obtained from four host-prediction tools (BLASTn, PHIST, VirHostMatcher-Net, and RaFAH).

File Name: Supplementary Data 9
Description: Correlations between composition of host classes, predicted for phages from all environments in IMG/VR and hosts predicted for aMGVs. All assignments are based on results from PHIST. Pearson's correlation coefficient (*r*) and *p*-value were calculated in SciPy 1.9.1 using two-tailed t distribution.

File Name: Supplementary Data 10
Description: Taxonomic assignments of 298 ancient metagenomic gut viruses (aMGVs) using geNomad tool based on collection of taxonomy marker genes (https://portal.nersc.gov/genomad/index.html).

File Name: Supplementary Data 11
Description: Clustering high-quality and complete ancient metagenomic gut viruses (aMGVs) into approximately genus- and family-level groups on the basis of pairwise average amino acid identity (AAI) and gene sharing following https://github.com/snayfach/MGV/tree/master/aai_cluster .

File Name: Supplementary Data 12
Description: vContact2 results for viral cluster with *Mushuvirus mushu* (NODE_310_length_36983_cov_28.516681). P-values were obtained from vContact2 output.

File Name: Supplementary Data 13
Description: Population microdiversity of complete bacteriophage genomes from modern Hadza hunter gatherers's gut from samples where Mushuvirus was identified (DOI: 10.1016/j.cell.2023.05.046). Calculation was performed using InStrain 1.8.0.

File Name: Supplementary Data 14
Description: Results of BLASTn search for Left_flank and Right_flank flanking regions (241 bp and 119 bp) of genome *Mushuvirus mushu* against Genome Taxonomy Database (GTDB) and the Unified Human Gastrointestinal Genome (UHGG) collection. Results indicate that species *ER4* from *Oscillospiraceae* family was a host for *Mushuvirus mushu* found in the 1300-year-old palaeofecal material. However, potential host-range was much broader (see Supplementary Data 11). ANI = Query Coverage Per Subject * Percentage of identical matches / 100.

File Name: Supplementary Data 15
Description: Broad host range of *Mushuvirus mushu* genome based on BLASTn search against Genome Taxonomy Database (GTDB) and the Unified Human Gastrointestinal Genome (UHGG) collection. ANI = Query Coverage Per Subject * Percentage of identical matches / 100.
