# Supplementary material for: Ultraconserved bacteriophage genome sequence identified in 1300-year-old human palaeofaeces
Source: Nat Commun. 2024 Jan 23;15:495. doi: 10.1038/s41467-023-44370-0 (PMC10805732; doi:10.1038/s41467-023-44370-0)
Supplement: Supplementary file 5 — Reporting Summary [file 41467_2023_44370_MOESM5_ESM.pdf]

nature portfolio

Corresponding author(s): Andrzej Zielezinski, Bas E. Dutilh

Last updated by author(s): Dec 4, 2023

Reporting Summary

Nature Portfolio wishes to improve the reproducibility of the work that we publish. This form provides structure for consistency and transparency in reporting. For further information on Nature Portfolio policies, see our Editorial Policies and the Editorial Policy Checklist.

Statistics

For all statistical analyses, confirm that the following items are present in the figure legend, table legend, main text, or Methods section.

n/a	Confirmed	
[ ]	[x]	The exact sample size (*n*) for each experimental group/condition, given as a discrete number and unit of measurement
[x]	[ ]	A statement on whether measurements were taken from distinct samples or whether the same sample was measured repeatedly
[ ]	[x]	The statistical test(s) used AND whether they are one- or two-sided<br>*Only common tests should be described solely by name; describe more complex techniques in the Methods section.*
[x]	[ ]	A description of all covariates tested
[x]	[ ]	A description of any assumptions or corrections, such as tests of normality and adjustment for multiple comparisons
[ ]	[x]	A full description of the statistical parameters including central tendency (e.g. means) or other basic estimates (e.g. regression coefficient) AND variation (e.g. standard deviation) or associated estimates of uncertainty (e.g. confidence intervals)
[ ]	[x]	For null hypothesis testing, the test statistic (e.g. *F*, *t*, *r*) with confidence intervals, effect sizes, degrees of freedom and *P* value noted<br>*Give P values as exact values whenever suitable.*
[x]	[ ]	For Bayesian analysis, information on the choice of priors and Markov chain Monte Carlo settings
[x]	[ ]	For hierarchical and complex designs, identification of the appropriate level for tests and full reporting of outcomes
[ ]	[x]	Estimates of effect sizes (e.g. Cohen's *d*, Pearson's *r*), indicating how they were calculated

Our web collection on statistics for biologists contains articles on many of the points above.

Software and code

Policy information about availability of computer code

Data collection	No software was used to collect data for this study. Downloaded datasets include:<br>- IMG/VR v.4<br>- GTDB v.07-RS207<br>- NCBI RefSeq release 215<br>- NCBI GenBank release 251<br>- PHROG v.4<br>- UHGG v.2.0<br>- Virus Metadata Resource release 12/02/2022 from ICTV
Data analysis	The source code used in this study allowed for reconstructing ancient bacteriophage genomes from human hut metagenomic samples. The source code was implemented as a pipeline developed using Snakemake and is available on GithHub (https://github.com/rozwalak/Ancient_gut_phages).<br><br>Other software used in this study:<br>1. Metagenomic sample preprocessing and genome assembly:<br>- Cutadapt v.4.1<br>- KneadData v.0.12.0<br>- Metaspades v.3.15.5<br>- Bowtie2 v.2.4.4<br>- SAMtools v.1.14

- Fastqc v.0.12.0
- PyDamage v.0.70

2. Virus identification:
   - Jaeger v.1.1.0,
   - VIBRANT v.1.2.1
   - VirSorter2 v.2.2.3
   - CheckV v.1.0.1
3. Host and lifestyle prediction
   - PHIST v.1.1.0
   - BLASTn v.2.13.0+
   - VirHostMatcher-Net v.1.0
   - RaFAH v.0.1
   - BACPHLIP v.0.9.6
4. Taxonomic classification:
   - vContact2 v.0.11.3
   - geNomad v.1.3.3
   - ViPTreeGen v.1.1.370
   - Cytoscape v.3.9.0
   - GraPhlAn v.1.1.3
5. Genome and proteome sequence comparisons:
   - VIRIDIC v.1.1
   - EzAAI v.1.2.2
   - InStrain v.1.8.0
   - Geneious Prime v.2023.04
   - BLASTp v.2.13.0+
   - DIAMOND v.2.0.15
   - HMMER v.3.3.2
   - MAFFT v.7.308
   - needle/water from EMBOSS v.6.6.0.0

For manuscripts utilizing custom algorithms or software that are central to the research but not yet described in published literature, software must be made available to editors and reviewers. We strongly encourage code deposition in a community repository (e.g. GitHub). See the Nature Portfolio guidelines for submitting code & software for further information.

Data

Policy information about availability of data

All manuscripts must include a data availability statement. This statement should provide the following information, where applicable:

- Accession codes, unique identifiers, or web links for publicly available datasets
- A description of any restrictions on data availability
- For clinical datasets or third party data, please ensure that the statement adheres to our policy

Ancient phage genome sequences and their gene annotations generated in this study have been deposited in the Zenodo database [https://doi.org/10.5281/zenodo.7919433]. The reconstructed ancient genome sequence of Mushuvirus mushu is available from NCBI GenBank under accession BK063464 [https://www.ncbi.nlm.nih.gov/nuccore/BK063464]. Supporting data generated in this study are provided in the Supplementary Information/Source Data. Accession numbers of ancient metagenomic samples used in this study are available in the AncientMetagenomeDir [https://github.com/SPAAM-community/AncientMetagenomeDir]. Other databases used in the study include: IMG/VR v.4 [https://genome.jgi.doe.gov/portal/IMG_VR/], GTDB v.07-RS207 [https://data.ace.uq.edu.au/public/gtdb/data/releases/release207/], UHGG v.2.0 [http://ftp.ebi.ac.uk/pub/databases/metagenomics/mgnify_genomes/human-gut/v2.0/], NCBI GenBank release 251 [https://www.ncbi.nlm.nih.gov/genbank/], NCBI RefSeq release 215 [https://www.ncbi.nlm.nih.gov/refseq/], PHROG v.4 [https://phrogs.lmge.uca.fr], and Virus Metadata Resource release 12/02/2022 from ICTV [https://ictv.global/vmr]. Source data are provided with this paper.

Research involving human participants, their data, or biological material

Policy information about studies with human participants or human data. See also policy information about sex, gender (identity/presentation), and sexual orientation and race, ethnicity and racism.

Reporting on sex and gender	N/A
Reporting on race, ethnicity, or other socially relevant groupings	N/A
Population characteristics	N/A
Recruitment	N/A
Ethics oversight	N/A

Note that full information on the approval of the study protocol must also be provided in the manuscript.

Field-specific reporting

Please select the one below that is the best fit for your research. If you are not sure, read the appropriate sections before making your selection.

[x] Life sciences [ ] Behavioural & social sciences [ ] Ecological, evolutionary & environmental sciences

For a reference copy of the document with all sections, see nature.com/documents/nr-reporting-summary-flat.pdf

Life sciences study design

All studies must disclose on these points even when the disclosure is negative.

Sample size	No sample-size calculation was conducted. The dataset included 72 ancient DNA metagenomic samples from human feces or digestive gut content, sourced from the European Nucleotide Archive (ENA). As all available samples were included, the sample size could not be increased. With 298 ancient bacteriophage genomes obtained, surpassing previous studies, the sample size was deemed adequate for the analysis.
Data exclusions	No data was excluded.
Replication	The study is computational in nature and relies on well-established algorithms and publicly available databases. The code for data analysis is scripted in a manner that allows for easy replication of the results and shared with the publication. All parameters used in the tools are explicitly stated in the Methods sections. This ensures that the study can be reproduced by other researchers. All analyses were reproduced at least twice in silico, always with a successful replication of the results.
Randomization	In this computational study, the allocation into groups is based on the inherent characteristics of the bacteriophage genomes and their derived protein sequences. The study does not involve random allocation or control of covariates as it would in a traditional experimental design. Instead, sequences are grouped based on computational analyses, such as clustering based on sequence similarity. Thus we have not assessed any statistical hypotheses other than these assumed in well-established bioinformatical tools, methods or previously reported evolution models. Therefore, the concept of random allocation or control of covariates is not applicable to this study.
Blinding	None of the analyses required random grouping or between group comparisons, so no blinding was needed. The analyses were performed on bacteriophage genomes and their derived protein sequences using automated computational methods. There was no subjective interpretation of data that could be influenced by knowledge of the sample identities, making the concept of blinding inapplicable in this context. All steps are clearly outlined and can be reproduced for verification.

Reporting for specific materials, systems and methods

We require information from authors about some types of materials, experimental systems and methods used in many studies. Here, indicate whether each material, system or method listed is relevant to your study. If you are not sure if a list item applies to your research, read the appropriate section before selecting a response.

Materials & experimental systems

n/a	Involved in the study	
[x]	[ ]	Antibodies
[x]	[ ]	Eukaryotic cell lines
[x]	[ ]	Palaeontology and archaeology
[x]	[ ]	Animals and other organisms
[x]	[ ]	Clinical data
[x]	[ ]	Dual use research of concern
[x]	[ ]	Plants

Methods

n/a	Involved in the study	
[x]	[ ]	ChIP-seq
[x]	[ ]	Flow cytometry
[x]	[ ]	MRI-based neuroimaging

Plants

Seed stocks	N/A
Novel plant genotypes	N/A
Authentication	N/A
